# The Effects of Lead Acetate on Sexual Behavior and the Level of Testosterone in Adult Male Rats

**Published:** 2011-03-21

**Authors:** Mokhtar Mokhtari, Maryam Zanboori

**Keywords:** Lead Acetate, Sexual Behavior, Testosterone, Rat

## Abstract

**Background:**

In the present study, the oral effect of lead acetate on the parameters related to sexual behavior as well as changes in the level of testosterone hormone in adult male rats have been investigated.

**Materials and Methods:**

Forty adult male Wistar rats were allocated into five equal groups. The control group received nothing, the sham group received distilled water and the experimental groups received 25, 50 and 100mg/kg lead acetate orally, respectively for 28 days. The changes in testosterone hormone level and following sexual behavior parameters were investigated: mount latency (ML), intromission latency (IL), post ejaculatory interval (PEI), mount frequency (MF), ejaculatory latency (EL), intromission frequency (IF), copulatory efficacy (CE) and intercopulatory interval (ICI).

**Results:**

The levels of testosterone hormone in the groups that received 50 and 100 mg/kg lead acetate showed significant decreases in compared to the control group. Additionally, the same doses of lead acetate caused significant increases in ML, IL, PEI and EL compared to the control group. No significant change was observed in MF, but a significant decrease was detected in IF and CE in the experimental group that received 100 mg/kg lead acetate when compared with the control group. ICI showed significant decreases in the experimental groups that received 50 and 100 mg/kg lead acetate compared to the control group.

**Conclusion:**

It can be concluded that ingestion of lead acetate affects some behavioral activities and the testosterone level of male rats. These effects might be conducted via the alteration of leydig cells following lead acetate poisoning.

## Introduction

The decrease in fertility and male reproduction is associated with certain special toxic chemicals in the environment. There is a close relationship between infertility and vocational toxicity. Lead is a heavy element in the environment. Its use, particularly in gasoline, has made it widespread. In addition, lead is a toxic element whose effects appear over a long time. The human body receives lead through food (65%), water (30%) and weather (15%) (1, 2); any organ or system in the body might be affected through the mechanisms involved in the main biochemical processes. These mechanisms include lead’s capability to control or mimic calcium function and its effect on proteins (3, 4).

Lead overexposure may cause anemia, renal disorders, reproductive abnormalities and neural conditions such as sudden seizures, behavior abnormalities or a low intelligence quotient (IQ). Even exposure to a low level of lead may bring about changes in the body’s physiological functions (5, 6). A strong mechanism of lead neurotoxicity is its ability to function as a calcium agonist in a few processes. Lead affects the zinc transcription factor area by binding to DNA and creating different responses (7). In addition, lead has a great tendency to bind to the calmodulin, a binding protein to calcium ions and thereby may affect cellular physiology such as signal transduction (8).

Studies show that the main target of lead toxicity is male fertility and the axis of pituitary-testis reproduction, thus causing infertility, reduction in sperm levels and morphological changes in people with excessive lead exposure (9).

The present study seeks to investigate the oral effects of lead acetate on the parameters related to sexual behavior in male rats which include: mount latency (ML), intromission latency (IL), post ejaculatory interval (PEI), mount frequency (MF), ejaculatory latency (EL), intromission frequency (IF), copulatory efficacy (CE), intercopulatory interval (ICI) and changes in the blood testosterone hormone level (10). By doing so, the results could be helpful in preventing lead overexposure and optimizing its use in industry.

## Materials and Methods

### Animals and treatment

The Ethics Committee at Islamic Azad University, Kazerun Branch has reviewed and approved ethical conciderations in the present study.

For the purpose of this study, male and female Wistar rats, supplied by the Animal Breeding Center of Islamic Azad University, Kazerun were used. Rats weighed 200-220 g and were 2.5-3 months old. They were maintained under standard conditions of light, food and temperature.

Since the observation related to parameters of sexual behavior should be performed at night, we darkened a room and placed all rats in that environment while reversing their day and night cycles. The observation and experiment took place in the same location. To observe the parameters of sexual behavior, a glass cage was placed on a tabletop one meter above the floor.

To observe the rats, a 5 watt red lamp was utilized. Light conditions were as follows: 7 am to 7 pm was considered the dark period and 7 pm to 7 am was the light period. Throughout the experiment, male and female rats were maintained separately except for behavioral observation when they were placed together in the glass cage.

Cages were washed and sterilized every other day. The environmental temperature was controlled at 22 ± 2°C (11, 12).

To ensure a limited weight range, the rats’ weights were measured prior to the experiment and male rats who weighed 200-220 g were selected. In total, there were 40 male rats divided into the following five groups: 1. control group (A) received only condensed food and water during the experiment with no solvent or medicine, 2. sham group (B) received 0.2 ml oral solvent (distilled water) in 24 hours, 3. first experimental group (C) received 25 mg/kg oral lead acetate in 24 hours, 4. second experimental group (D) received 50 mg/kg oral lead acetate in 24 hours, and 5. third experimental group (E) received 100 mg/kg oral lead acetate in 24 hours (8). All experimental groups were administered lead acetate (MERK, Germany) for 28 days. Since recording the sexual behavior was impossible outdoors, therefore a glass cage was provided based on a sample presented by Mendelson and Gorzalka (10).

### Preparation of animals

#### Preparation of males

Before performing the experiments, active and inactive males were separated. A male rat had 25 minutes to copulate a female rat (intromission or ejaculation). The male rats that were unable to perform the copulation within this period were separated from the other males. Studies have shown that male rats with no sexual experience exhibit increases in the IL, EL and PEI periods.

Thus, in order to obtain sexual experience each male rat copulated several times prior to the main observations with prepared females.

#### Preparation of females

Almost 60 adult female Wistar rats were used on different days. Preparation of female rats was identical in the experimental, control and sham groups.

All female rats were maintained in separation several times before the experience in the glass cage for adaptation. To induce sexual admittance, progesterone (50 μg) and estradiol valerate (100 μg) were used. Forty-eight hours prior to observation, each female rat was injected subcutaneously with 100 μg of estradiol valerate dissolved in olive oil. Female rats that underwent experimentation were left untreated for the next 48 hours. Following the experiment, 1 ml white alcohol was injected into the female rat vagina to prevent pregnancy and kill the sperm.

#### Drug administration and records of parameters related to sexual behavior

An insulin syringe with a feeder was used for drug injections that were administered every morning at a given time. Having injected the 25, 50 and 100 mg/kg lead acetate, parameters related to sexual behaviors were recorded through per second observations for 120 minutes. The following parameters were recorded: mount latency (ML) or the interval between placing a female animal near a male animal and the first copulation attempt. Copulation is the behavior in which the male animal is mounted on the back of the female. Intromission latency (IL): the interval between placing a female animal near a male animal and the first male intromission. Intromission is a copulatory behavior in which the male animal genital organ enters the female vagina. Ejaculatory latency (EL): the interval between placing a female animal near a male with ejaculation. In ejaculation, which is often accompanied by intromission, the male animal mounts on the back of female animal. The male genital organ enters the female vagina rapidly and stays there for a moment. Rapid and rhythmic movements can be observed on the back of the male animal.

It is worth noting that the intromission accompanied by ejaculation is longer than the usual and common intromissions. Additionally, the pressure that the male animal exerts into the female animal is greater in this condition. Post ejaculatory interval (PEI): the interval between the ejaculation and the first next copulation intromission. In this period, the male animal is separated from the female counterpart. Mount frequency (MF): the number of times that the male animal copulates with the female animal without intromission. MF is calculated in the copulation series before ejaculation. Intromission frequency (IF) refers to the number of times that the male copulates with intromission and performs this action in a copulation series before ejaculation.

Copulatory efficacy (CE) is not obtained by observation, but is measured by the formula $\mathrm{CE}=\frac{\mathrm{IF}}{\mathrm{MF}+\mathrm{IF}}$

It shows that copulation is completely successful. Intercopulatory interval (ICI) is the interval between two copulations accompanied by intromission and is calculated by the following formula: $\mathrm{ICI}=\frac{\mathrm{IF}}{\mathrm{EL}}$(10).

We measured each of the above mentioned factors in different copulatory series.

A copulatory series is the interval between the first male animal activities for copulation until ejaculation, followed by their separation.

During the observation time (about 120 minutes for every male animal), several copulation series were observed and factors were determined by indices. After the 28 day treatment with lead acetate, blood samples obtained from the rats’ hearts were collected in test tubes under mild ether anesthesia at the end of day 28.

After about 15 minutes, test tubes that contained blood samples were gathered and placed in an incubator at 37°C for 30 minutes.

After coagulation the tubes were placed into the centrifuge at 5000 rpm. Sera was pipetted by Pasteur pipettes and transferred into new, labeled tubes sealed with parafilm and stored frozen until the measurement of testosterone by RIA.

#### Statistical analysis

 SPSS was used from data analysis. ANOVA analyzed means. In case the means were different, TUKEY test was used for comparison of pairs. Each mean value was compared with the control group. P≤0.05 was considered significant.

## Results

**Fig 1:**
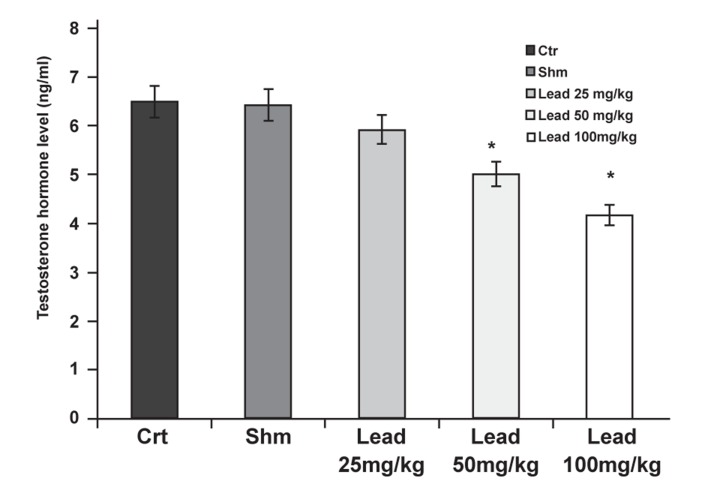
The effects of different doses of lead acetate on testosterone hormone levels in the experimental and control groups (*p≤0.05).

The investigation into the effects of different doses of lead acetate on the concentration of testosterone indicated that the groups who received 50 and 100 mg/kg lead acetate significantly decreased (p≤0.05) when compared with the control group (Fig 1).

**Fig 2:**
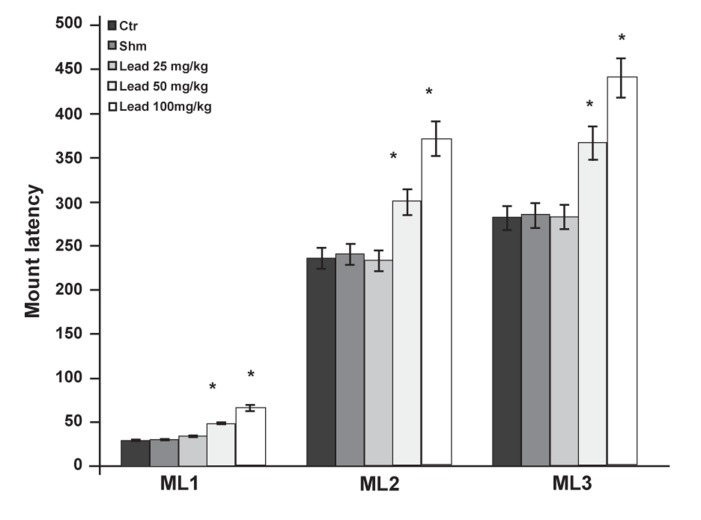
Comparison of ML factor between the experimental groups receiving different doses of lead acetate and control group in the first (ML1), second (ML2) and third (ML3) copulatory series (*p≤0.05).

The effects of different doses of lead acetate on ML in different series (ML1, ML2, ML3), indicated a significant increase (p≤0.05) in the groups that received doses of 50 and 100 mg/kg lead acetate compared to the controls (Fig 2).

**Fig 3:**
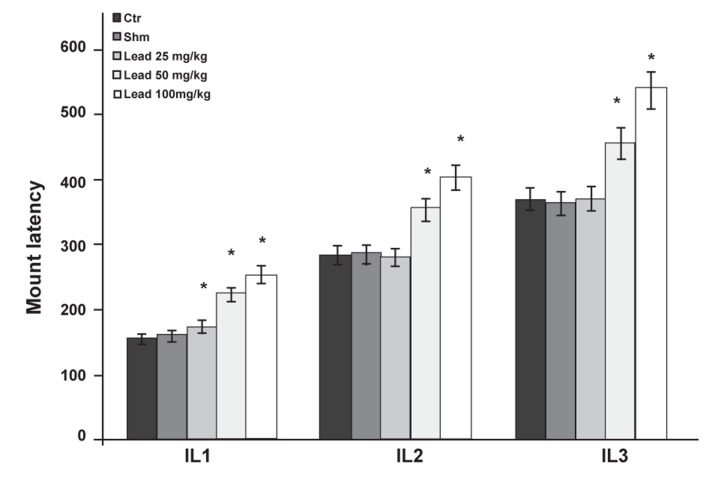
Comparison of IL factor between the experimental groups receiving different doses of lead acetate and control group in first (IL1), second (IL2) and third (IL3) copulatory series (*p≤0.05).

The investigation of the effects of different doses of lead acetate on IL in different copulation series showed that IL1 in the first copulation series in the groups receiving 25, 50 and 100 mg/kg lead acetate significantly increased when compared with the control group. In the second and third copulation series, factors IL2 and IL3 showed significant increases in the experimental group (50 and 100 mg/kg) compared to the control group (p≤0.05) but not in the group that received 25 mg/kg lead acetate (Fig 3).

**Fig 4:**
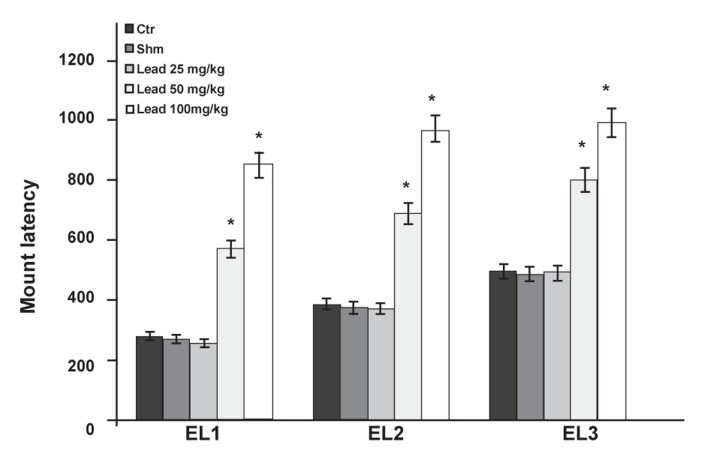
Comparison of EL factor between the experimental groups receiving different doses of lead acetate and control group in first (EL1), second (EL2) and third (EL3) copulatory series (*p≤0.05).

Statistical test results related to factor EL in the EL1, EL2 and EL3 copulatory series show that the groups which received 50 and 100 mg/kg of lead acetate had significant increases (p≤0.05) when compared with the control group (Fig 4).

**Fig 5:**
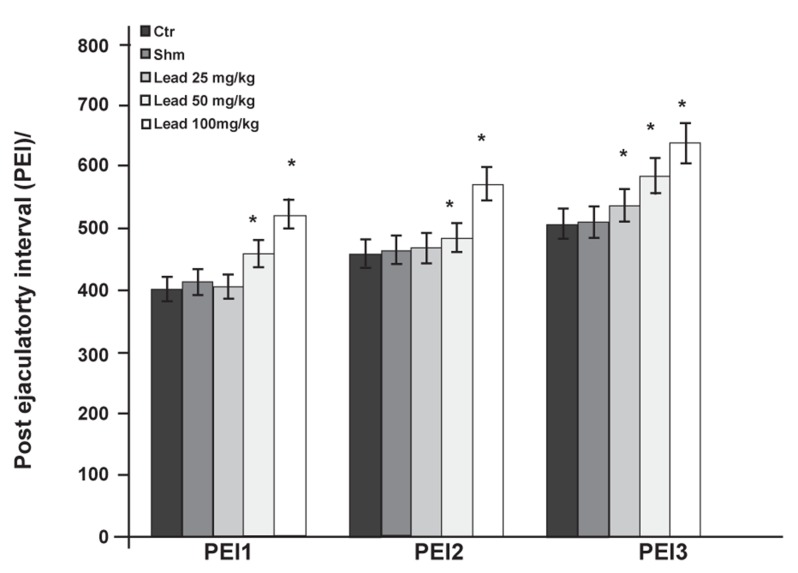
Comparison of PEI factor between the experimental groups receiving different doses of lead acetate and control group in first (PEI1), second (PEI2) and third (PEI3) copulatory series (*p≤0.05).

The results related to PEI in the first and second copulation series showed significant increases (p≤0.05) in the experimental groups that received 50 and 100 mg/kg lead acetate than the control group (Fig 5).

**Fig 6:**
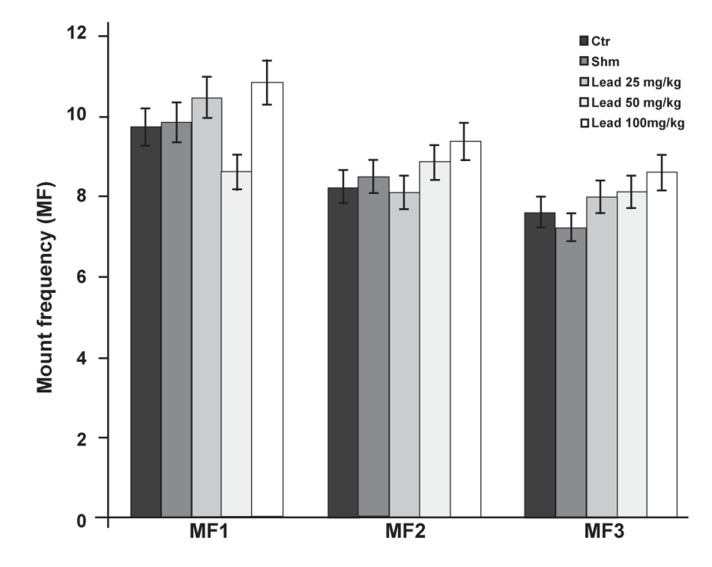
Comparison of MF factor between the experimental groups receiving different doses of lead acetate and the control group in first (MF1), second (MF2) and third (MF3) copulatory series (*p≤0.05).

The investigation of lead acetate effect on the MF,series was not significantly different (p≤0.05) among all the experimental groups and the control group (Fig 6).

**Fig 7:**
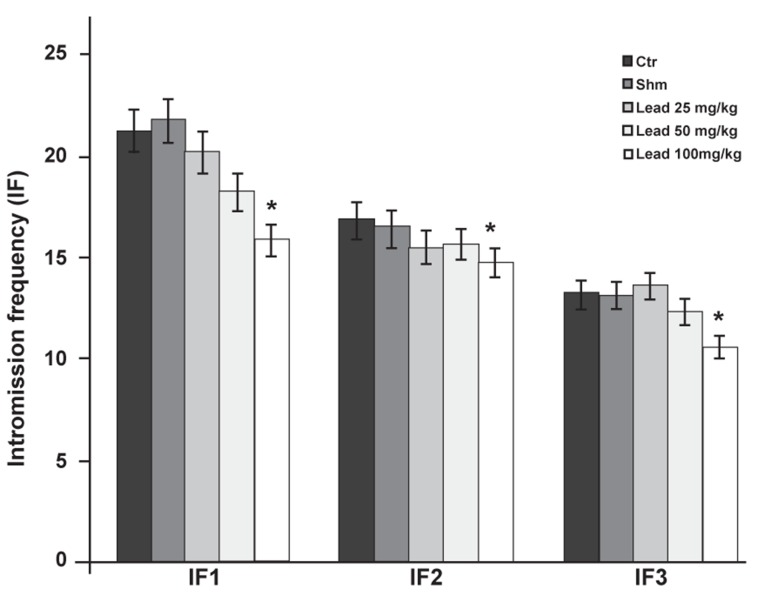
Comparison of IF factor between the experimental groups receiving different doses of lead acetate and control group in first (IF1), second (IF2) and third (IF3) copulatory series (*p≤0.05).

A comparison of statistical test results related to IF factors (IF1, IF2 and IF3) between the experimental groups that received 25 and 50 mg/kg lead acetate and the control group did not show a significant decrease (p≤0.05), however the experimental group that received the 100 mg/kg dose significantly decreased (p≤0.05) compared to the control group (Fig 7).

**Fig 8:**
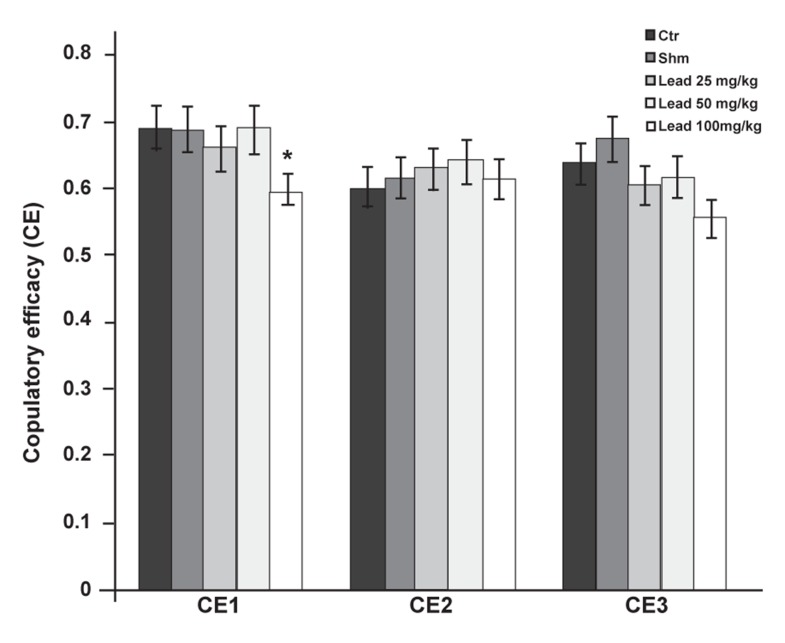
Comparison of CE factor between the experimental groups receiving different doses of lead acetate and control group in first (CE1), second (CE2) and third (CE3) copulatory series (*p≤0.05).

Also, statistical comparison of the results related to CE1 showed that the group which received 100 mg/kg lead acetate significantly decreased (p≤0.05) compared to the controls. No significant difference was observed between the experimental groups administered 25 and 50 mg/kg lead acetate, and the control group. In the second and third copulation series, no significant difference in CE parameters was observed between the groups receiving doses of 25, 50 and 100 mg/kg lead acetate and controls (Fig 8).

**Fig 9:**
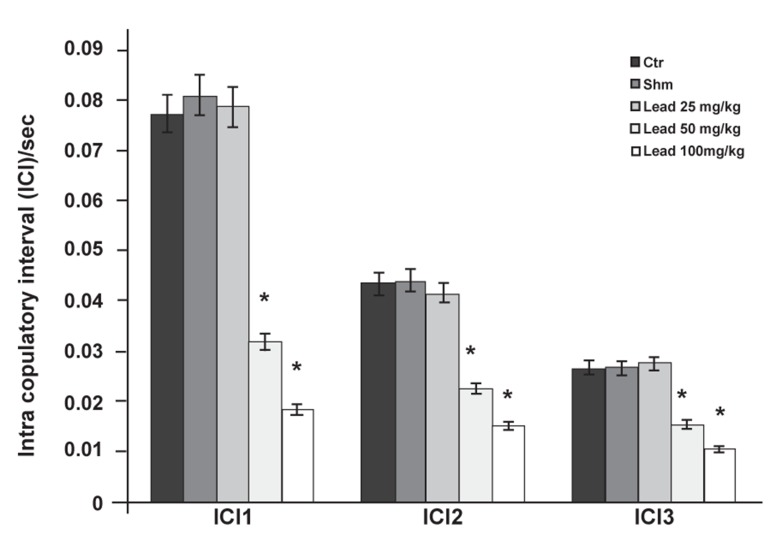
Comparison of ICI factor between the experimental groups receiving different lead (*p≤0.05).

Statistical comparison of the results related to ICI parameter (ICI1, ICI2, ICI3) indicated a significant decrease (p≤0.05) in the groups administered 50 and 100 mg/kg lead acetate compared to the control group, however in the group that received 25 mg/kg lead acetate, no significant difference was observed compared to the control group (Fig 9).

acetate levels and control group in first (ICI1), second (ICI2) and third (ICI3) copulatory series.

## Discussion

Decreases in fertility and reproduction are associated with toxic chemicals in the environment; there is a close correlation between infertility and vocational toxicity (9). Lead is a heavy metal that leaves harmful effects on testis tissue, fertility and reproduction in males, particularly humans (13).

Considering the present findings, testosterone hormone levels in the experimental groups that received 50 and 100 mg/kg lead acetate significantly decreased compared to the control group (Fig 1). Accordingly, probably lead toxicity caused a defect in the pituitary and hypothalamus in luteinizing hormone (LH) secretion, direct damage in the testis seminiferous tubules and decreased testosterone hormone secretion from leydig cells (14- 16). Besides, studies have shown that lead affects testis steroidogenic activity, as well as testosterone and gonadotropin serum levels in albino rats (17). Lead acetate administered during 14 days caused a decrease in testis weight and sexual appending organs. Also, it decreased steroidogenic enzymes such as Δ5 -3 β-HSD, 17β-HSD activity testis, LH, follicle stimulating hormone (FSH) level and testosterone (17). In the present study, decreased testosterone hormone could be in agreement with decreased in steroidogenic enzyme functions. Results have shown that cholesterol-protein binding strength decreases because of the effect of lead and it is clear that the first cholesterol metabolism phase is disturbed because of this effect (18). Since cholesterol is the precursor of testosterone synthesis, its decrease causes decreased testosterone synthesis consistent with the obtained results. Lead’s negative effects on progesterone and testosterone involve a decrease in cytochrome P450Scc, p450c17 and 17 β-HSD in the biosynthesis of steroid hormones (19). The reported studies have shown that lead salts can increase angiotensine II by causing a disorder in the sodium-potassium pump (20, 21). Angiotensine II, by connecting to the available LH receptors in the leydig cell membranes, causes inhibition of adenylate cycle activity, inhibition of cAMP construction and testosterone hormone level decreases due to Leydig cells (22). Evidence shows that parameters indirectly indicating sexual motivation and sexual performance are as follows: ML, IL and PEI. Lead acetate effect on ML showed that this chemical at doses of 50 and 100 mg/kg increased ML (Fig 2). Research findings have also shown that neurotransmitters are involved in such processes as sexual motivation and sense of smell. Lead causes some changes in the catecholaminergic system, particularly the dopaminergic system (11), which is necessary for sexual behavior. Lead decreases dopamine production due to the effect on dopaminergic and serotonergic systems and increases ML (23, 24). Other studies have shown that the rat brain’s preoptic area is the site of gonad steroids that modulate sexual behavior and gonadotropin secretion (25). Probably lead acetate decreases norepinephrine (NE) and dopamine from the preoptic area and increases ML.

The IL factor indicates intromission latency. Groups that received doses of 25, 50 and 100 mg/ kg lead acetate showed significantly increased IL in the first copulatory series. However in the second and third copulatory series, this increase was observed in IL only at doses of 50 and 100 mg/ kg (Fig 3). Nitric oxide synthase (NOS) may be a target for lead and change in its function causes pathophysiologic effects cascade (26).

As previously mentioned, lead decrease the level of dopamine level in the preoptic area. Dopamine mediates the performance of and in fact, erection is the precense of nitric oxide (NO) (27, 28). Considering the erection function mechanism and sildenafil function in erection improvement, it could be said that lead causes a disorder in this mechanism by decreasing NO synthesis. Disorders in erectile function caused a disorder in intromission of the groups that received 50 and 100 mg/kg lead acetate during the first and second copulatory series that had significantly increased PEI (Fig 5). In the third copulatory series, all experimental groups significantly increased.

The neurosteroid production from adrenal and gonadal steroid endocrine sources may affect male sexual behavior through GABAA receptors. This receptor is located in the preoptic area. It is essential and necessary for sexual attraction, intercourse, erection and ejaculation. These steroid gonadal sources occur in the brain through testosterone aromatization, which is essential for sexual behavior and sensitivity. Neurosteroids also may modulate activities related to smell in order to sexually attract males (29). Considering the negative effect of lead on P450 (19) steroidogenic acute regulatory protein (STAR) (30), cytochrome enzyme, it is suggested that these enzymes activities decrease to produce a neurosteroid and cause the decrease of an essential neurosteroid to stimulate sexual behavior and function.

Decreased enzymatic activities in the olfactory bulb cause disturbances in smell-related behaviors and decreases in sexual function (31, 32). The inhibiting effect of lead on NE results in the decrease in the level of GnRH which in turn causes a decrease in gonadotropin and testosterone, and finally a reduced sexual stimulation and function (33). Lead acetate has decreasing effects on testis steroidogenic function, gonadotropin serum and testosterone levels (14, 34). Since the presence of testosterone hormone is required for sexual performance, its decrease causes this parameter decrease. No significant difference has been found between the groups that received doses of 25, 50 and 100 mg/kg lead acetate in the MF copulatory series and the control group (Fig 6). Groups that received 50 and 100 mg/kg lead acetate in copulatory series two and three had significantly increased EL (Fig 4) and PEI compared to the control group (Fig 5). The 100 mg/kg group in the copulatory series showed a significant decrease in IF (Fig 7). Ejaculation is affected by neural and hormonal factors. Lead blocks N-methyl-D-aspartate (NMDA) receptors and inhibits calcium flow (35). It also decreases NMDA receptor phosphorylation, which consequently causes a defect in male sexual behavior and increases EL.

The groups that received 25 and 50 mg/kg lead acetate had no significant change in CE when compared with the control group, whereas the group that received 100 mg/kg lead acetate showed a significant decrease only in the first copulatory series (Fig 8).

As mentioned before, lead acetate leaves an unsuitable effect on copulatory efficacy and causes a disorder in sexual function by decreasing the mentioned parameters, in particular IF. Long term consumption of industrial metal salts such as magnesium sulfate, aluminum chloride, lead acetate and lead chloride create adverse effects on sexual behavior, fertility and the adult male rat reproductive system. It also decreases copulatory efficacy (36). The obtained results show that ICI factors in the groups that received 50 and 100 mg/kg lead acetate were significantly decreased in all three copulatory series compared to the controls (Fig 9).

## Conclusion

We may conclude that 50 and 100 mg/kg lead acetate increases ML, IL, PEI and EL parameters and causes a disorder in sexual motivation and performance. In addition, 100 mg/kg lead acetate causes a significant decrease in IF and CE parameters which is indicative of a disorder in copulation. Lead acetate causes decreased sexual motivation and reproductive activities due to the decreased testosterone hormone secretion, which is probably due to its effect on the central nervous system and disordered regulation and secretion of neurotransmitters.
